# Cancer-Associated Fibroblast Proteins as Potential Targets against Colorectal Cancers

**DOI:** 10.3390/cancers16183158

**Published:** 2024-09-14

**Authors:** Ruchi Shah, Katherine A. Johnson, Anna E. L. Lippert, Sean G. Kraus, Philip B. Emmerich, Cheri A. Pasch, Wei Zhang, Kristina A. Matkowskyj, Aaron M. LeBeau, Dustin A. Deming

**Affiliations:** 1 Division of Hematology, Medical Oncology and Palliative Care, Department of Medicine, School of Medicine and Public Health, University of Wisconsin, Madison, WI 53726, USA; 2 McArdle Laboratory for Cancer Research, Department of Oncology, University of Wisconsin, Madison, WI 53705, USA; 3 Carbone Cancer Center, University of Wisconsin, Madison, WI 53705, USA; 4 Department of Pathology and Laboratory Medicine, The University of Kansas Medical Center, Kansas City, KS 66160, USA; 5 Department of Pathology and Laboratory Medicine, School of Medicine and Public Health, University of Wisconsin, Madison, WI 53726, USA; 6 Department of Radiology, School of Medicine and Public Health, University of Wisconsin, Madison, WI 53706, USA

**Keywords:** fibroblast activation protein, colorectal cancer, liver metastasis, cancer-associated fibroblasts, tumor markers, immunohistochemistry

## Abstract

**Simple Summary:**

Due to the lack of cancer cell-specific markers in colorectal cancer (CRC), the utility of antibody-mediated therapies in this disease setting has been limited. Here, we evaluated whether markers of cancer-associated fibroblasts (CAFs) may more specifically target CRCs. We identified fibroblast activation protein (FAP) as a strong candidate for targeting CRC. FAP is highly expressed in a majority of cancers and is absent in over 90% of normal tissues. These data indicate that FAP may be a strong candidate for antibody conjugate therapy and support the continued development of FAP-targeting conjugate therapies. Additionally, we identified associations between CAF marker co-expression and immunostimulatory pathways. CAF markers might be useful as targets for immune engaging therapies, with combinations of CAF markers holding promise to identify those patients most likely to respond. Overall, these data provide the basis for potential further studies investigating the utility of CAF markers to target CRCs.

**Abstract:**

In colorectal cancer (CRC), attempts to identify cancer cell-specific markers to guide antibody-mediated therapeutics have failed to uncover markers that are both exclusive to cancer tissues and abundant across CRCs. Alternatively, cancer-associated fibroblasts (CAFs), which are abundant in the tumor microenvironment and upregulate unique surface markers, are not found in healthy tissues. Here, we evaluated the expression patterns of CAF-associated proteins α-smooth muscle actin (αSMA), fibroblast activation protein (FAP), podoplanin (PDPN), matrix metalloproteinase-2 (MMP2), transgelin (TAGLN), and THY1. While αSMA and THY1 were abundant in cancer tissues, high abundance in normal tissues limited their targeting potential. FAP was present in 94.5% of primary and metastatic CRC tissues and absent in 93.7% of adjacent normal colon and liver tissues assessed. These results indicate that FAP is a promising target for antibody conjugates with potential for broad application in CRC. Co-expression analyses showed that CRCs simultaneously expressing high levels of PDPN, MMP2, and THY1 were enriched for immune-related signatures, indicating potential for antibody-mediated immune engagers. Overall, this work highlights the potential of CAF proteins to act as therapeutic targets for novel anticancer agents and become important therapeutic biomarkers.

## 1. Introduction

Despite advances in treatment and screening, CRC remains a leading cause of cancer-related death [1]. Current treatment options for non-resectable metastatic (m) CRC include cytotoxic chemotherapy, targeted therapies for cancers with specific molecular alterations, or immunotherapy for patients with mismatch-repair deficient (dMMR) cancers [2]. However, outside of an immune checkpoint blockade for dMMR cancers, the durability of the benefit of these treatments is limited, and thus, novel therapeutics are urgently needed for patients with mCRC. Antibody-mediated therapies, including those bound to cytotoxic agents, radioisotopes, or immune engagers, are of particular interest. However, these therapies require a cancer-specific target.

Due to the heterogeneous nature of CRC and the development of these cancers from normal intestinal cells, identification of cell surface targets that can distinguish cancer cells from normal epithelial cells has proven to be a major challenge [3,4]. Cancer-associated fibroblasts (CAFs) are a key component of the TME and are known to promote metastasis, angiogenesis, immunosuppression, and therapeutic resistance through signaling and crosstalk with other TME components and cancer cells [5]. As CAFs have phenotypes not often seen in normal tissues, CAF-associated processes and proteins may be potential targets for cancer therapies. The THY1 (CD90) cell surface antigen is often used as a pan-CAF marker, especially in CRC [6,7]. Additionally, alpha-smooth muscle actin (αSMA), transgelin (TAGLN), fibroblast activation protein (FAP), podoplanin (PDPN), and matrix metalloproteinase-2 (MMP2) have been associated with distinct functional subtypes of CAFs [6]. While these proteins may mark distinct individual cells, many are associated with high expression in tumors overall. The expression of αSMA has long been associated with the onset of CRC [8]. Additionally, FAP is highly expressed across several solid tumors [9,10]. High expression of these markers in CRC indicates they may be promising targets for antibody–drug conjugate therapy, but a focused analysis on their expression patterns across tumor and normal tissue and subtypes of disease is necessary. Many markers associated with CAFs are not exclusive to CAFs, either. FAP has been shown to be expressed on cancer cells themselves, which increases the likelihood of these proteins effectively targeting cancer [11].

In addition to antibody-delivered cytotoxic therapy, the development of immune engagers has been an exciting new direction for antibody-mediated therapies. Immune engagers most often refer to bi-specific antibodies that engage a tumor cell and a CD3+ T cell to facilitate T cell recognition [12,13,14]. However, research has expanded to novel targets on both immune cells and tumor cells to broaden the mechanisms by which immune cell behavior in the TME can be altered [12,14]. Because of their role in immune regulation [5], CAFs may be important potential targets for these novel therapies.

Here, we evaluated several CAF markers for their potential as therapeutic targets for CRC for the delivery of cytotoxic chemotherapy or as adjuncts to immunotherapy. We identified 239 CRC patients spanning all stages of disease. Histological sections of both tumor and adjacent normal samples from these patients were evaluated for the expression of the CAF markers THY1, αSMA, TAGLN, FAP, PDPN, and MMP2. Additionally, collagen levels were evaluated as a proxy for overall stromal content of the tumor. We found that FAP was expressed highly across tumor tissue and rarely in normal colon or liver tissues, indicating it as a strong target candidate. Overall, our analyses indicate that further research should focus on developing FAP-targeted antibody conjugates as treatments for CRC. Additionally, we observed that high expression of specific combinations of these targets relate to altered expression of immune-related pathways, highlighting both the potential for CAFs to be good targets for antibody-guided immune adjuncts and the need for further characterization of CAF marker functions in combination.

## 2. Materials and Methods

### 2.1. Tissue Samples

A total of 243 CRC patients spanning all stages of disease, 90 of which have mCRC to the liver, were sampled. For 239 of these patients, 1–2 samples of primary cancer tissue and 1 sample of adjacent normal colon tissue was available and used for further analyses. An additional sample from the metastatic cancer site and an adjacent normal liver sample were also evaluated for the 90 mCRC patients.

### 2.2. Immunohistochemistry

Tissue sections were deparaffinized and rehydrated using standard methods. Antigen retrieval was performed using citrate buffer for FAP, αSMA, PDPN, TAGLN, THY1, and MMP2, and EDTA buffer for CD8. The endogenous peroxidase activity was quenched using Peroxidazed 1 (BioCare Medical, Pacheco, CA, USA) followed by blocking with Background Sniper (BioCare Medical). The following primary antibodies were used: FAP (E1V9V) (Cell Signaling Technologies (CST), Danvers, MA, USA, #66562) in PBS at 1:200, αSMA (D4K9N; CST #19245) in PBS at 1:250, PDPN (LpMab-12; CST #26981) in PBS at 1:250, TAGLN (polyclonal; Abcam, Cambridge, UK, #ab155272) in PBS at 1:1000, THY1/CD90 (D3V8A; CST #13801) in PBS at 1:500, and MMP2 (D4M2N; CST #40994) in PBS at 1:100. Trichrome staining for collagen was performed using the trichrome stain kit (Abcam #150686). The mismatch repair status was assessed as previously described using markers for MLH1, MSH2, MSH6, and PMS27. CD8 staining was performed at the UW Health Surgical Pathology laboratory on a BenchMark ULTRA automated system. Briefly, antigen retrieval was performed for 64 min in buffer CC1 (Roche, Indianapolis, IN, USA), and the primary antibody (CD8 clone SP57, Roche #790-4460) was incubated for 20 min and detected with Ventana UltraView Universal DAB Buffer (Roche #760-500).

### 2.3. Sequencing of Mutations

DNA from formalin-fixed paraffin-embedded (FFPE) samples was isolated on a Maxwell 16 AS2000 or Maxwell CSC (Promega, Madison, WI, USA) using a Maxwell DNA LEV Plus DNA Kit (Promega #AS1135) or a Maxwell CSC FFPE DNA Kit (Promega #333515). DNA isolated from each patient’s samples were sequenced with the Qiagen Comprehensive Cancer Panel (Qiagen, Hilden, Germany, #33515) and sequenced on Illumina HiSeq 2500 or NovaSeq 6000. Sequencing analyses were performed through the UW Biotechnology Center. Clean-up of reads was performed through quality trimming via Skewer [15] and alignment to Homo sapiens build 1k_v37 was performed using BWA-MEM [16]. Deduplication was performed through Picard (https://picard.sourceforge.net; accessed on 3 September 2017) and Je [17]. Base quality scores were calibrated using GATK [18] and mutations were called using Strelka v-2.8.4 [19] without matched controls and then annotated using SnpEff [20]. These VCF files were uploaded to Galaxy at usegalaxy.org [21] and cross-referenced to ClinVar’s database via their downloadable VCF (accessed on 29 April 2019) for annotation of predicted clinical response. If ClinVar’s database labeled a variant as “Pathogenic” or “Likely Pathogenic”, it was categorized as a pathogenic variant. Mutations in *APC*, *KRAS*, *BRAF*, *PIK3CA*, and *TP53* were further analyzed for possible variants not in ClinVar.

### 2.4. Scoring Tissue Sections

Stromal fibroblast staining was analyzed by two observers. Scores were assigned using a binning system based on stromal staining intensity and prevalence: 0 for no staining, 1 for low or weak staining, 2 for moderate staining, and 3 for abundant and intense staining. CD8^+^ T cell staining was quantified as the number of tumor-infiltrating lymphocytes (TILs) within neoplastic epithelium per high power field (HPF; 40× objective). Tissue samples were deemed non-scorable if tissue was detached or folded.

### 2.5. TCGA Data Analysis

TCGA PanCancer Atlas COADREAD cohort data [22,23] were accessed via cbioportal.org [24]. Marker-high expressing groups were defined by a Z-score cutoff of EXP > 1 for each marker (αSMA, FAP, MMP2, PDPN, TAGLN, and THY1). Overlap for groups was calculated using cbioportal.com. Differential gene expression for each group with five or greater CRCs vs. the remaining COADREAD dataset was accessed and downloaded for further analysis. Some upset plots were adapted from cbioportal.com.

### 2.6. Gene Set Enrichment Analysis

Gene Set Enrichment Analysis (GSEA) was performed using the differential gene expression between each selected intersecting or individual marker expressing group and the remaining cohort from the TCGA PanCancer Atlas COADREAD dataset [22,23]. GSEA was run against the Hallmark Gene Sets using GSEA software version 4.2.3 [25], using the Run GSEA PreRanked function. Genes were ranked by significance of differential expression using the formula −log10(*p* value) × (sign of fold change). Heatmaps were built in R version 4.3.0 [26] using the package ComplexHeatmap version 2.16 [27,28].

### 2.7. Statistical Methods

Data were analyzed using R (version 4.1.0) [26] to obtain frequencies, distributions, and summary statistics. Wilcoxon rank sum tests, Pearson correlation tests, and Chi-square tests were run where indicated. For all tests, *p* value < 0.05 was considered significant.

## 3. Results

### 3.1. Expression of CAF Markers in Cancerous Tissue

To determine the utility of different CAF markers in targeting cancer tissue, we identified a cohort of 243 patients, each with one or two colon cancer tissue samples, giving us a total of 483 primary cancer samples. Of these 243, 239 patients also had matched adjacent normal colon available for histological analysis. Additionally, 90 of these 239 patients also had 1–2 liver metastasis samples and 1 matched normal liver tissue sample, totaling 175 mCRC samples and 90 matched normal liver samples. Full demographic information can be found in Supplementary Material, Table S1.

**Figure 1:**
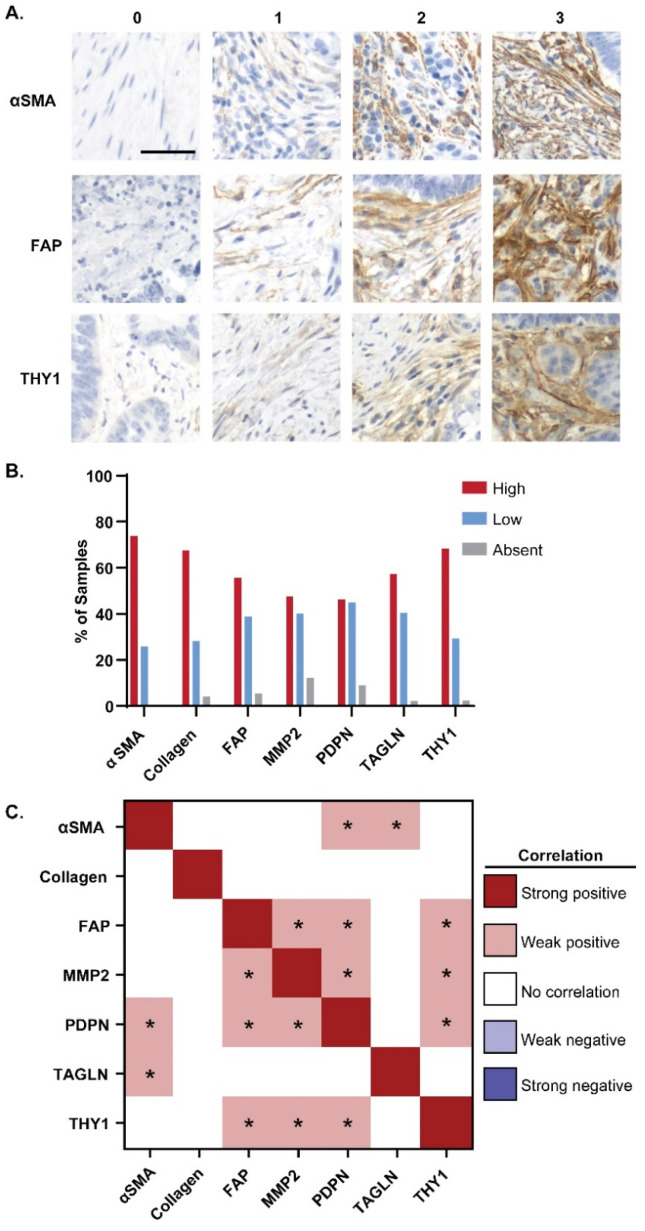
CAF marker expression in CRC patient tissues. Tissue microarrays with patient CRC tissues were stained with CAF markers and scored 0–3+. (**A**) Representative staining and scoring for αSMA, FAP, and THY1 in CRC tissues across tissue microarrays. Scale bar = 50 µm. (**B**) Frequency of marker-absent (score 0), marker-low (score 1), and marker-high (score 2–3) CRC tissue samples for each CAF marker (αSMA, collagen, FAP, MMP2, PDPN, TAGLN, and THY1) (**C**) Correlations between marker expression scores across all CRC tissues were evaluated by Pearson’s Correlation tests performed on all pairs of CAF markers. Strength of correlation was binned by correlation coefficient, where a correlation coefficient (*r*) ≥ 0.6 indicated a strong positive correlation, 0.3 ≤ *r* < 0.6 indicated a weak positive correlation, −0.3 ≤ *r* < 0.3 indicated no correlation, −0.6 ≤ *r* < −0.3 indicated a weak negative (inverse) correlation, and *r* < −0.6 indicated a strong negative (inverse) correlation. Asterisks denote statistical significance (*p* < 0.05).

To identify CAF markers that could potentially target CRCs, we performed immunohistochemistry (IHC) analyses to measure the abundance of αSMA, FAP, MMP2, PDPN, TAGLN, and THY1 (Figure 1 and Supplementary Material, Figure S1) [6]. Masson’s trichrome stain was used to evaluate collagen abundance and thereby assess the overall abundance of stromal tissue within the TME. Marker expression in CRC tissue was scored from 0 to 3+. Representative staining and scoring for αSMA, FAP, and THY1 are shown in Figure 1A, and the remaining markers are shown in Supplementary Material, Figure S1. Samples scored as 0 are considered negatively staining or marker-absent, samples scored as 1+ are considered marker-low and samples scored as 2 or 3+ are considered marker-high. Additionally, samples scoring greater than or equal to 1 are considered positively staining or marker-present.

The most frequent highly expressed markers in CRC samples were αSMA (73.9%), THY1 (68.3%), and collagen (67.5%; Figure 1B). Other highly expressed markers included TAGLN at 57.2% and FAP at 55.8%. MMP2 and PDPN were highly expressed in less than half of cancers. Correlations between marker expression scores across all cancer samples were investigated to identify whether any of the selected markers could be found in the same populations (Figure 1C). After correlation coefficients were binned, no strong positive or negative correlations were identified across all markers and all CRC samples (Figure 1C). However, several statistically significant (*p* < 0.05) weak positive correlations were identified amongst CAF markers. Significant weak positive correlations were found pairwise across FAP, PDPN, MMP2, and THY1 (Figure 1C). Similar significant weak positive correlations were seen pairwise across αSMA, PDPN, and TAGLN (Figure 1C). PDPN showed a significantly weak positive correlation with the expression of the largest number of other CAF markers (Figure 1C). Overall, these data indicate strong expression of CAF markers in cancer tissues but heterogeneous expression across individual cancers.

### 3.2. Expression of CAF Markers in Normal Tissue

**Figure 2:**
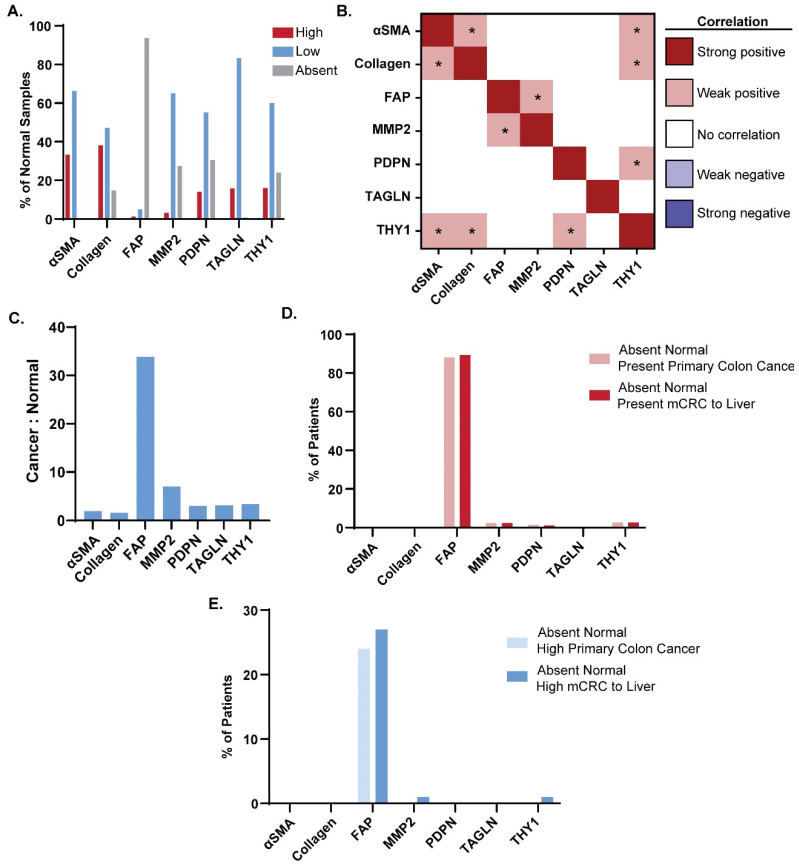
FAP expression specifically labels CRC tissue. (**A**) Frequency of marker-absent (score 0), marker-low (score 1), and marker-high (score 2–3+) in CRC patient adjacent normal tissue samples for each CAF marker (αSMA, collagen, FAP, MMP2, PDPN, TAGLN, and THY1). (**B**) Correlations between marker expression scores in adjacent normal tissues across all CRC patients were evaluated by Pearson’s Correlation tests performed between all pairs of CAF markers. Strength of correlation was binned by correlation coefficient as in Figure 1C. Asterisks denote statistical significance (*p* < 0.05) (**C**) Ratio of the number of patients with marker-high cancer tissues to the number of patients with marker-high adjacent normal. For each marker, only patients with scorable cancer and normal tissues were considered (αSMA: *n* = 118, collagen: *n* = 155, FAP: *n* = 123, MMP2: *n* = 132, PDPN: *n* = 121, TAGLN: *n* = 109, THY1: *n* = 123) (**D**) Percentage of mCRC patients with negative staining in adjacent normal colon and liver tissue that had positive-staining colon cancer (pink) or metastases to liver (red). (**E**) Percentage of mCRC patients with negative staining in adjacent normal colon and liver normal tissue that had high staining in colon cancer (light blue) or liver metastases (dark blue).

For antibody-mediated therapies to be tolerated as therapeutic agents, targets must have high expression in cancer tissues and low expression in normal tissues. FAP had the greatest frequency of marker-absent normal tissues across both colon and liver samples (93.7%; Figure 2A) while less than 40% of all normal samples stained negatively for each of the other markers (αSMA, THY1, TAGLN, MMP2, collagen, and PDPN). Correlations between marker expression scores across all normal tissue samples were also evaluated (Figure 2B). In normal tissues, no strong positive or negative correlations were observed across the CAF markers surveyed. A statistically significant (*p* < 0.05) weak positive correlation was identified pairwise across αSMA, collagen, and THY1 (Figure 2B). Similar to the cancer tissue samples, a significant weak positive correlation was also seen between MMP2 and PDPN and between THY1 and PDPN in adjacent normal tissues (Figure 2B).

### 3.3. Cancer-Specific Marker Expression

FAP was expressed at detectable levels in 94.5% of all CRC samples (Figure 1B) and absent in 93.7% of normal colon and liver samples (Figure 2A). At the individual marker level, the relationship between marker expression score in all primary cancer and adjacent primary normal tissue was considered (Supplementary Material, Figure S2). FAP and MMP2 had the greatest percentage of patients with marker-present primary cancer tissue and marker-absent adjacent normal tissues (88.5% and 89.8%, respectively), indicating that these markers can specifically label cancer tissue. To further characterize the cancer specificity of FAP, the correlation between marker expression scores in all metastases and adjacent normal liver tissue was considered for each individual marker (Supplementary Material, Figure S3). Based on this analysis, FAP had the highest proportion of scorable patients with marker-present cancer and marker-absent adjacent normal tissue (91.4%). This further emphasizes FAP’s specificity for CRC tissues, even in the context of liver metastases.

To more directly assess which markers exhibit the highest cancer tissue expression and the lowest normal tissue expression, the ratio between the number of marker-high cancer samples and marker-high normal samples was calculated (Figure 2C). FAP exhibited the highest ratio at 33.8, followed by MMP2 at 7.0. THY1, TAGLN, PDPN, αSMA, and collagen followed with ratios falling below 5.0 (Figure 2C). This established FAP and, to a lesser degree, MMP2 as the markers labeling CRC tissue with the greatest specificity.

When considering the cancer-specificity of a marker, it is imperative that the expression pattern of high expression in numerous cancer tissues and minimal expression in numerous tissues is reflected for individual patients. As such, we next evaluated how often patients with negative staining in normal tissues had positive or high expression of each of the markers evaluated. For these analyses, we calculated the percentage of mCRC patients (*n* = 90) with marker-absent adjacent normal tissues (colon and liver) with positive staining in primary or metastatic cancer tissues (Figure 2D). A total of 88.2% of patients with no FAP staining in both of their normal tissues had positively staining primary tumors, and 89.3% of patients with negative staining in both normal tissues had positively staining metastatic tissue. For all other CAF markers, less than 3% of all patients with negative staining in both normal tissues were marker-present in their primary or metastatic cancer tissue. FAP was also the target with the largest percentage of patients with marker-high cancer tissue and marker-absent colon and liver normal tissues at 24% and 27%, respectively (Figure 2E). These data indicate that FAP is the most promising target for CRC antibody-directed therapies, as it is minimally expressed in normal tissues but highly expressed in cancer tissues in both primary and metastatic disease. Furthermore, a high proportion of patients not expressing FAP in normal tissues had expression in cancer tissues, with almost one-quarter of cancers expressing this marker to a high degree, demonstrating that this target is highly specific in individual patients.

### 3.4. FAP Expression across Clinical Disease Subtypes

**Figure 3:**
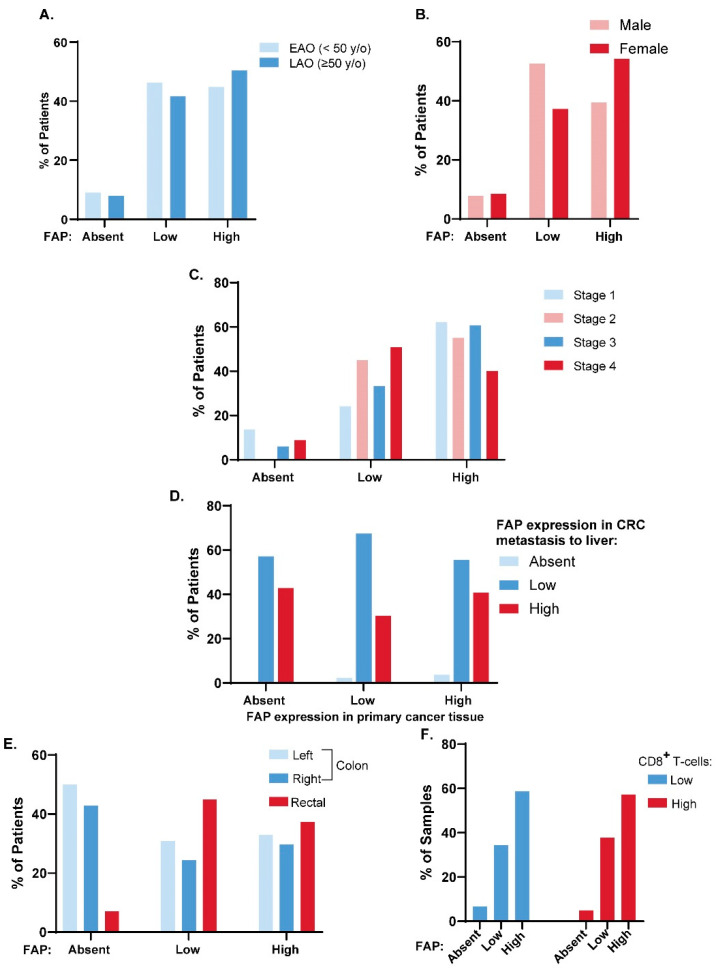
FAP expression across clinical subtypes of CRC. (**A**) Proportion of patients with EAO (light blue) or LAO (dark blue) disease and scorable FAP-absent, FAP-low, and FAP-high primary cancer tissues. (**B**) Proportion of male (pink) or female (red) patients with scorable FAP-absent, FAP-low, and FAP-high primary cancer tissues. (**C**) Proportion of patients with scorable FAP-absent, FAP-low, and FAP-high primary cancer tissues across CRC disease stage. (**D**) Proportion of patients with FAP-absent, -low, and -high liver metastasis tissue given absent, low, or high FAP expression in their primary cancer tissue. (**E**) Primary cancer tissue FAP expression (absent, low, and high) across patients with left-sided, right-sided, and rectal primary tumors (**F**) FAP expression (absent, low, and high) across all CRC samples with high CD8^+^ T cell infiltration (CD8^+^ TILs > 5/HPF) compared to those with low CD8^+^ T cell infiltration (CD8^+^ TILs < 5/HPF).

To determine whether there is a patient population for which FAP-targeted therapy may be of most benefit, we evaluated the expression of FAP across clinical characteristics of CRC (Figure 3). First, we determined whether there was a difference in FAP expression between patients with early-age onset disease (EAO; diagnosed before age 50) and late-age onset disease (LAO; diagnosed at age 50 or later). No significant difference in FAP expression was found between these two age cohorts (Figure 3A). The correlation between gender (Figure 3B) and stage (Figure 3C) with FAP expression was also evaluated, and no significant differences were identified. Next, the FAP expression in primary versus liver metastases was evaluated in matched primary and metastatic cancers (Figure 3D). The patients considered here had cancer at both primary and metastatic sites which were scorable for FAP expression (*n* = 77). Of patients whose primary cancers stained positively for FAP expression (*n* = 70), 97.1% also expressed FAP in their metastasis. In cases in which there was absent staining in primary cancers (*n* = 7), there was positive staining in metastatic lesions; thus, none of the patients tested had absent staining in both primary and metastatic lesions. In comparing primary tumor site, significantly higher FAP expression was observed in rectal cancers compared to colon cancers (Χ^2^ test: *p* = 0.037; Figure 3E). A significantly larger proportion of patients with scorable FAP-absent cancer tissues had primary tumors in the colon, with 50.0% of patients having left-sided colon tumors and 42.9% of patients having right-sided colon tumors (Figure 3E).

### 3.5. FAP Expression and CD8^+^ T Cell Tumor Infiltration

To evaluate if FAP expression correlated with a CD8^+^ T cell infiltrated cancer phenotype, IHC for CD8 was performed and scored as the number of CD8^+^ cells infiltrating the epithelial compartment (tumor infiltrating lymphocytes (TILs) per high powered field (HPF)). Tissues were defined as having high CD8^+^ T cell infiltration if they had greater than or equal to 5 CD8^+^ TILs/HPF and low CD8^+^ T cell infiltration if they had fewer than 5 CD8^+^ TILs/HPF [29]. The relationship between FAP score and CD8^+^ T cell infiltration is presented in Figure 3F. No significant difference in the prevalence of CD8^+^ T cells between FAP-absent, FAP-low, and FAP-high cancers was observed (Χ^2^ test: *p* = 0.764).

### 3.6. FAP Expression across Molecular Disease Subtypes

**Figure 4:**
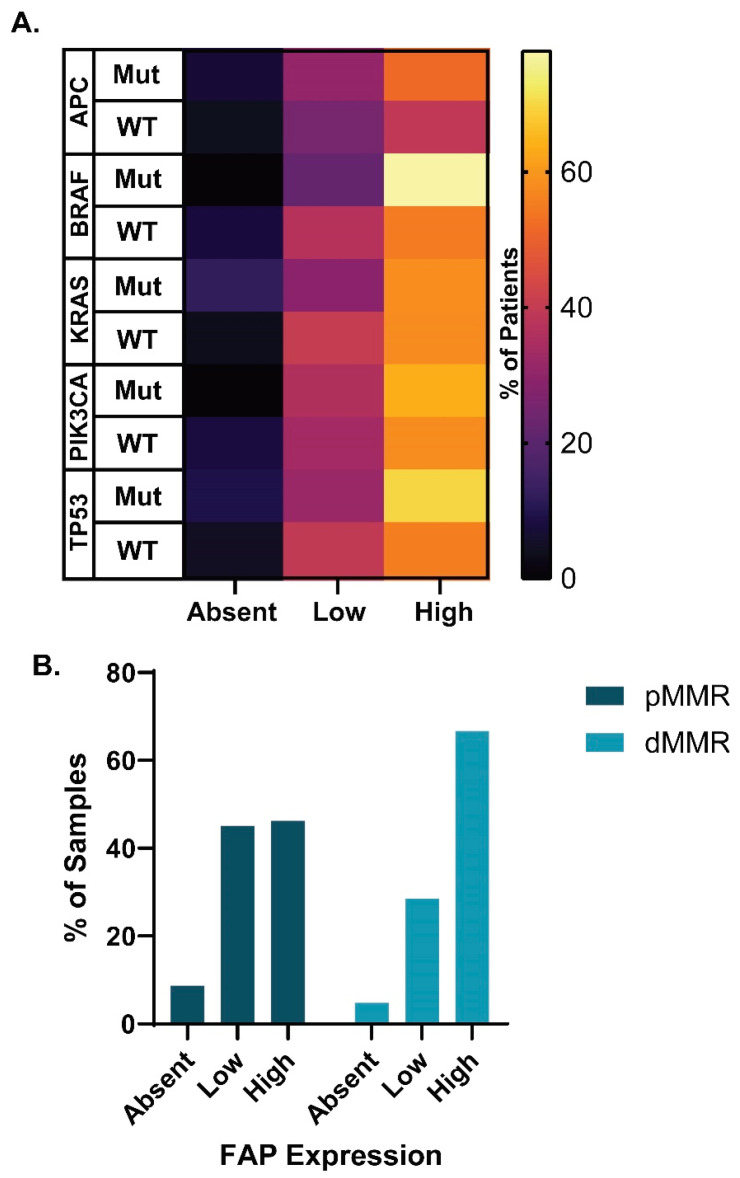
FAP expression across molecular CRC subtypes. (**A**) FAP expression in primary cancers tissues across *APC*, *BRAF*, *KRAS*, *PIK3CA*, and *TP53* mutant and wild-type CRCs. (**B**) Percentage of dMMR and pMMR cancers with FAP-absent, -low, and -high primary cancers.

The *APC*, *BRAF*, *KRAS*, *PIK3CA*, and *TP53* mutation status was assessed to determine if FAP was differentially expressed in cancers based on their mutational profile. No significant relationship was found between FAP expression and *APC*, *BRAF*, *KRAS*, *PIK3CA*, or *TP53* mutation status (Χ^2^ test: *p* = 0.749, 0.387, 0.097, 0.593, and 0.339, respectively; Figure 4A). Mismatch-repair (MMR) deficiency, a requirement for immunotherapeutic treatment and predictor of patient response in mCRC patients [30,31], was also assessed. No significant relationship was found between proficient MMR (pMMR) versus dMMR patients and FAP expression (Χ^2^ test: *p* = 0.208; Figure 4B).

### 3.7. CAF Marker Expression and CD8^+^ T Cell Infiltration

**Figure 5:**
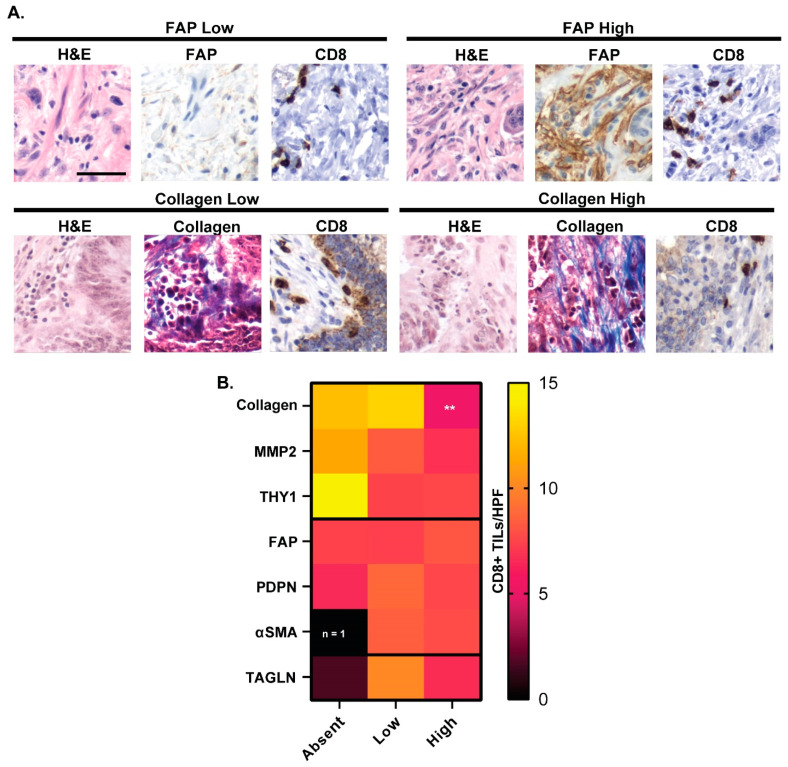
CD8^+^ T cell infiltration varies with collagen abundance in the CRC TME. (**A**) Representative CD8^+^ T cell staining in FAP-low and FAP-high expressing primary CRC (top row). Representative CD8^+^ T cell staining in collagen-low and collagen-high expressing primary CRC (bottom row). (**B**) CD8^+^ TILs/HPF across CAF marker expression in all CRC tissues. ** *p* < 0.01 according to Wilcoxon rank sum test.

While no significant association was found between FAP expression and CD8^+^ T cell infiltration, the relationship between other CAF marker expression and CD8^+^ T cell infiltration was evaluated (Figure 5), as markers whose expression coincides with CD8^+^ T cell infiltration could potentially serve as promising adjuncts for CRC immunotherapies. Representative staining of CD8^+^ TILs/HPF for FAP and collagen marker-high and low CRC tissues are shown in Figure 5A. The statistical significance between the average number of CD8^+^ TILs/HPF for each CAF marker compared to the average number of CD8^+^ TILs/HPF in each respective marker-absent tissue sample was assessed with Mann–Whitney analyses (Figure 5B). The only statistically significant difference in average CD8^+^ TILs/HPF was found between collagen-high and collagen-absent CRC tissues, where collagen-high cancers had a mean of 5.2 CD8^+^ TILs/HPF versus collagen-absent cancers having a mean of 12.3 CD8^+^ TILs/HPF (*p* = 0.004) (Figure 5B).

### 3.8. CAF Marker Expression and Upregulated Pathways in Cancer

**Figure 6:**
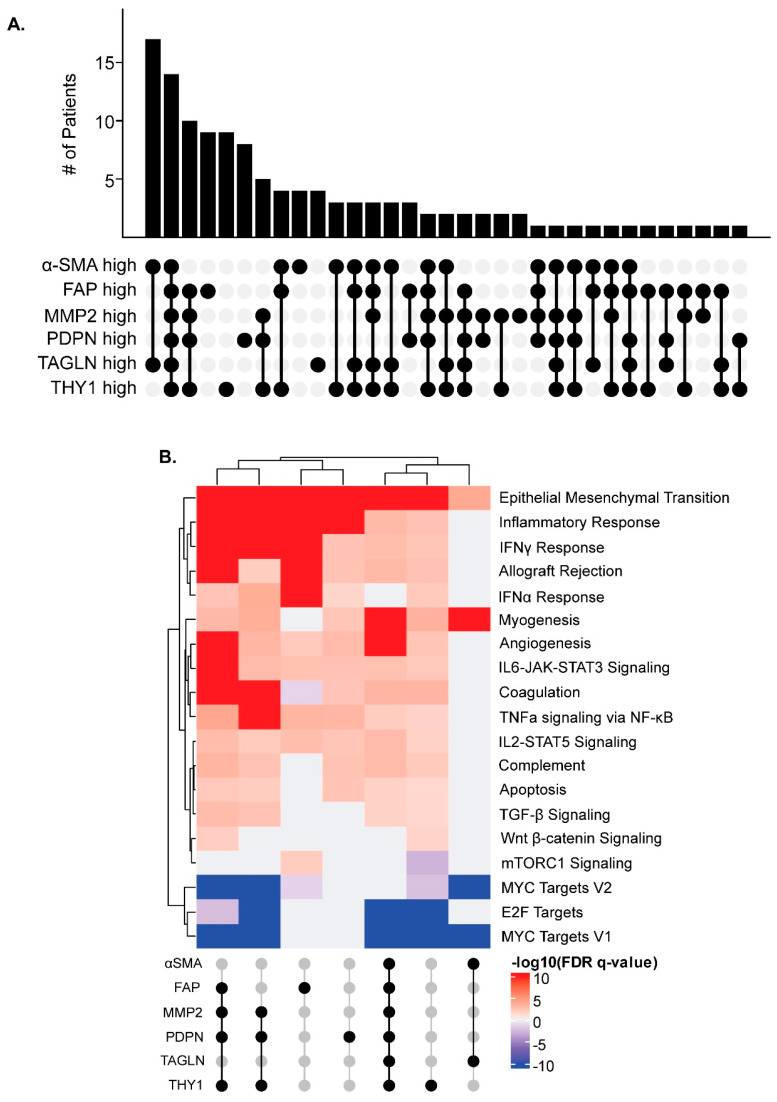
CAF markers are often co-expressed and associated with upregulation of inflammatory gene sets. (**A**) Groups of COADREAD cohort CRCs with intersecting and individual CAF marker expression. (**B**) GSEA comparing differential gene expression between intersecting or individual groups containing at least 5 CRCs vs. all other CRCs within the COADREAD dataset. Heatmap indicates –log10(FDR *q*-value) for each hallmark gene set by each group, where only statistically significant FDR q-values (*q* ≤ 0.05) are reported. Those groups with an FDR *q*-value > 0.05 for any given gene set were assigned a –log10(FDR *q*-value) of 0.

Since individual CAF markers did not predict T cell infiltration, combinations of markers were evaluated. We utilized the TCGA COADREAD dataset to identify CRCs with high RNA expression of each marker. Since collagen is encoded by several genes, it was not included in this analysis. We first evaluated the co-expression of CAF markers within CRCs. The most common overlap in marker expression was in CRCs exclusively upregulating αSMA and TAGLN together, followed by those CRCs simultaneously upregulating all markers evaluated (Figure 6A). Groups of CRCs with overlapping marker expression were further investigated using gene set enrichment analysis (GSEA) if five or more CRCs were included in the group. These groups included CRCs upregulating MMP2, PDPN, and THY1 together with or without upregulation of FAP, and those with individually upregulated FAP, THY1, and PDPN (Figure 6A). GSEA was utilized to evaluate the differential gene expression of each group of CRCs compared to all other CRCs in the COADREAD cohort. Consistent with known functions of CAFs, pathways generally upregulated across these groups included those related to contractility and extracellular matrix production, such as epithelial to mesenchymal transition and myogenesis, as well as inflammatory pathways, such as interferon gamma response and IL6-JAK-STAT3 signaling (Figure 6B). MYC and E2F target genes were consistently downregulated across these groups (Figure 6B). While some variation was seen between different groups, trends in up- and downregulated gene sets were largely consistent. Overall, high expression of CAF-related genes was associated with upregulation of similar pathways, regardless of which combination of CAF markers were upregulated.

## 4. Discussion

CAFs exhibit expression of proteins rarely observed in normal tissues [8], making them a promising candidate for cancer-specific antibody-mediated therapies. Ideal target proteins have high expression in cancer tissues and low or absent expression in normal tissues in the context of both primary and metastatic disease. Identifying associations between target expression and molecular disease subtypes or clinical features is imperative to determine if there is an ideal patient population for targeted conjugate therapy. We evaluated several CAF markers for their expression in primary and metastatic CRC and in the normal intestine and liver. Additionally, we evaluated the correlation of these potential targets with clinical and molecular characteristics. Furthermore, we assessed the relationship between potential target markers and the immune landscape to determine whether there may be benefit for use with immune engagers.

We identified FAP as a strong candidate for CRC-targeting cytotoxic treatments. While we found αSMA, collagen, and THY1 were most abundantly expressed at high levels in cancer tissues, these markers were also more commonly expressed in normal tissues, indicating increased potential for treatment-related adverse events if patients were treated with an αSMA-, collagen-, or THY1-targeting therapy. MMP2 was expressed at very low levels in normal tissue and highly expressed in 47.7% of cancer samples. FAP was expressed highly by 55.8% of cancer samples and was nearly absent in 93% of normal colon and liver samples. FAP is known to be expressed by stromal fibroblasts in the TME of many epithelial cancers [9,10]. Additionally, FAP has been found to be expressed on some CRC cancer cells, adding to its potential as a cancer target [11]. FAP expression has also been found to be associated with lower survival, especially in mCRC [32]. This study supports previous literature identifying FAP as a potential target in CRC [33,34,35,36].

Studies evaluating the use of a FAP targeting antibody alone to potentially deplete FAP^+^ cells have had limited to no anti-tumor efficacy in clinical trials [37,38]. Given these past results FAP-targeting antibodies are being studied in conjunction with cytotoxic chemotherapy or radiotherapeutics, that may affect adjacent cancer cells. Preclinical studies have shown the feasibility of this method [39,40]. It is imperative that future studies focus on the potential benefits of increasing cytotoxic or radiotherapeutic drug concentrations near cancer cells through FAP targeting versus the costs of potentially depleting FAP^+^ stromal cells as a result of using FAP-targeting antibodies. Fortunately, early preclinical work appears to support that such antibody–drug conjugates may more selectively kill cancer cells than slower-growing FAP^+^ stromal cells [39]. FAP-targeting antibodies and inhibitors are also being evaluated as potential conjugates for imaging. There are numerous ongoing clinical trials for the use of radiolabeled FAP-targeting antibodies as imaging agents, and a few studies are also exploring replacing radiolabeling with a radiotherapeutic agent [41,42]. We found that FAP is not only high in primary cancers and low in normal colon tissue but also highly expressed within metastases to the liver but not adjacent normal liver tissue. This finding adds to the value of FAP as a target, as it can also potentially be used to target metastatic disease.

Overall, FAP expression does not appear to be exclusive to any CRC clinical characteristics, including age, gender, and stage of disease. We did observe higher expression of FAP in patients with rectal cancers compared to colon cancers. We also found that FAP expression in primary tumors usually correlated FAP presence in liver metastases, though not completely. No significant relationship between molecular profile and FAP expression was found.

While we found little to no FAP expression in adjacent normal cells in our study, the expression of FAP in other normal tissues as well as non-cancerous diseased tissues needs to be evaluated to ensure that FAP targeting has no off-target effects. FAP-based imaging technologies are also being studied in cases of fibrosis, including in the lung [43]. Understanding the role of FAP in other fibrotic diseases will be important as these technologies emerge to ensure proper patient selection.

While our studies point towards FAP as an ideal general target for CRC due to its low expression in normal tissues, many of the markers evaluated still display relatively low expression in normal tissues and high expression in cancer tissues. As such, they may be useful as targets in certain disease settings, such as immunotherapy adjuncts. To explore this possibility, we first evaluated whether any of these markers were associated with CD8^+^ T cell infiltration, which has implications for immunotherapy response. However, only collagen was associated with altered CD8^+^ T cell infiltration, despite previous RNA-based reports that FAP is associated with an immune suppressive environment [44] We next expanded our search to identify altered pathways in marker-high cancers. We found that identifying associations between individual markers and biological processes is complex, as many of these markers are often co-expressed to a high degree in tumors. Those cancers with high expression of PDPN, MMP2 and THY1 were enriched for immune-mediated pathways, such as inflammatory response, IFN response, and IL6-JAK-STAT signaling. This indicates that these cancers could be more likely to benefit from immunotherapy approaches, but further investigation is required. Furthermore, the lack of association with CD8^+^ T cells and singular markers may indicate that multiple markers in tandem, perhaps related to overall functions, may be better indicators of tumor microenvironment states, which will benefit the most from immune targeting.

The generation of antibody-conjugated therapies with cytotoxic, radioisotope, or immunotherapy payloads are of great clinical interest for the treatment of cancer. This work identifies FAP as a highly promising TME protein for antibody-based therapies targeting CRC secondary to its expression across a wide range of these cancers and limited expression in the liver and colon. Further research using human and mouse cross-reactive FAP antibodies, such as the one developed by Hintz et al. [45], will aid with evaluating antibody-drug conjugates in in vivo models that can then be translated to patients.

## 5. Conclusions

The studies presented herein demonstrate the efficacy of FAP as a cancer-specific marker and highlight the potential of FAP and other CAF markers as targets for antibody-conjugated therapies or immune-adjuncts. In addition to being highly expressed in primary and metastatic CRC tissues, FAP is absent in the vast majority of normal tissues. Additionally, FAP expression patterns in liver metastases of CRCs are consistent with those of the patient’s primary cancers, indicating the utility of FAP-targeting therapies in both metastatic and non-metastatic patients. FAP expression in CRCs is correlated with cancers of the rectum, however no other clinical or molecular characteristics were found to correlate with FAP expression. As such, antibody-conjugated therapies targeting FAP could potentially target CRCs across a large population of CRC patients. Additionally, we found that co-expression of other CAF markers, MMP2, PDPN, and THY1, in CRC tissues was associated with upregulation of immune related pathways, meaning this co-expression pattern could potentially serve as a marker for patient response to immunotherapy. Further in vivo studies are necessary to confirm the efficacy of antibody-conjugated therapies targeting FAP and determine if CAF markers can potentially predict immunotherapy response.

## Data Availability

The original contributions presented in the study are included in the article/supplementary material, further inquiries can be directed to the corresponding author/s.

## Supplementary Materials

The following supporting information can be downloaded at: https://www.mdpi.com/article/10.3390/cancers16183158/s1, Figure S1. Representative staining and scoring for collagen, MMP2, PDPN, and TAGLN in CRC patient tissues across tissue microarrays. Figure S2. Heatmaps depicting percentage of all CRC patients with marker expression score in their primary CRC and marker expression in adjacent normal colon. Figure S3. Heatmaps depicting percentage of all mCRC patients with a marker expression score in their CRC metastases to liver and marker expression in adjacent normal liver. Table S1. Patient demographics.

## References

[1] The American Cancer Society Key Statistics for Colorectal Cancer. https://www.cancer.org/cancer/types/colon-rectal-cancer/about/key-statistics.html.

[2] Benson A.B., Venook A.P., Al-Hawary M.M., Arain M.A., Chen Y.-J., Ciombor K.K., Cohen S., Cooper H.S., Deming D., Farkas L. (2021). Colon Cancer, Version 2.2021, NCCN Clinical Practice Guidelines in Oncology. J. Natl. Compr. Cancer Netw..

[3] Abbasian M., Mousavi E., Arab-Bafrani Z., Sahebkar A. (2019). The Most Reliable Surface Marker for the Identification of Colorectal Cancer Stem-like Cells: A Systematic Review and Meta-Analysis. J. Cell. Physiol..

[4] Belov L., Zhou J., Christopherson R.I. (2011). Cell Surface Markers in Colorectal Cancer Prognosis. Int. J. Mol. Sci..

[5] Sahai E., Astsaturov I., Cukierman E., DeNardo D.G., Egeblad M., Evans R.M., Fearon D., Greten F.R., Hingorani S.R., Hunter T. (2020). A Framework for Advancing Our Understanding of Cancer-Associated Fibroblasts. Nat. Rev. Cancer.

[6] Nurmik M., Ullmann P., Rodriguez F., Haan S., Letellier E. (2020). In Search of Definitions: Cancer-Associated Fibroblasts and Their Markers. Int. J. Cancer.

[7] Lee H.O., Hong Y., Etlioglu H.E., Cho Y.B., Pomella V., Van den Bosch B., Vanhecke J., Verbandt S., Hong H., Min J.W. (2020). Lineage-Dependent Gene Expression Programs Influence the Immune Landscape of Colorectal Cancer. Nat. Genet..

[8] Tlsty T.D., Hein P.W. (2001). Know Thy Neighbor: Stromal Cells Can Contribute Oncogenic Signals. Curr. Opin. Genet. Dev..

[9] LeBeau A.M., Brennen W.N., Aggarwal S., Denmeade S.R. (2009). Targeting the Cancer Stroma with a Fibroblast Activation Protein-Activated Promelittin Protoxin. Mol. Cancer Ther..

[10] Garin-Chesa P., Old L.J., Rettig W.J. (1990). Cell Surface Glycoprotein of Reactive Stromal Fibroblasts as a Potential Antibody Target in Human Epithelial Cancers. Proc. Natl. Acad. Sci. USA.

[11] Yuan Z., Hu H., Zhu Y., Zhang W., Fang Q., Qiao T., Ma T., Wang M., Huang R., Tang Q. (2021). Colorectal Cancer Cell Intrinsic Fibroblast Activation Protein Alpha Binds to Enolase1 and Activates NF-ΚB Pathway to Promote Metastasis. Cell Death Dis..

[12] Rolin C., Zimmer J., Seguin-Devaux C. (2024). Bridging the Gap with Multispecific Immune Cell Engagers in Cancer and Infectious Diseases. Cell Mol. Immunol..

[13] Tapia-Galisteo A., Sánchez Rodríguez Í., Aguilar-Sopeña O., Harwood S.L., Narbona J., Ferreras Gutierrez M., Navarro R., Martín-García L., Corbacho C., Compte M. (2022). Trispecific T-Cell Engagers for Dual Tumor-Targeting of Colorectal Cancer. Oncoimmunology.

[14] Fenis A., Demaria O., Gauthier L., Vivier E., Narni-Mancinelli E. (2024). New Immune Cell Engagers for Cancer Immunotherapy. Nat. Rev. Immunol..

[15] Jiang H., Lei R., Ding S.W., Zhu S. (2014). Skewer: A Fast and Accurate Adapter Trimmer for next-Generation Sequencing Paired-End Reads. BMC Bioinform..

[16] Li H. (2013). Aligning Sequence Reads, Clone Sequences and Assembly Contigs with BWA-MEM. arXiv.

[17] Girardot C., Scholtalbers J., Sauer S., Su S.Y., Furlong E.E.M. (2016). Je, a Versatile Suite to Handle Multiplexed NGS Libraries with Unique Molecular Identifiers. BMC Bioinform..

[18] McKenna A., Hanna M., Banks E., Sivachenko A., Cibulskis K., Kernytsky A., Garimella K., Altshuler D., Gabriel S., Daly M. (2010). The Genome Analysis Toolkit: A MapReduce Framework for Analyzing next-Generation DNA Sequencing Data. Genome Res..

[19] Kim S., Scheffler K., Halpern A.L., Bekritsky M.A., Noh E., Källberg M., Chen X., Kim Y., Beyter D., Krusche P. (2018). Strelka2: Fast and Accurate Calling of Germline and Somatic Variants. Nat. Methods.

[20] Cingolani P., Platts A., Wang L.L., Coon M., Nguyen T., Wang L., Land S.J., Lu X., Ruden D.M. (2012). A Program for Annotating and Predicting the Effects of Single Nucleotide Polymorphisms, SnpEff: SNPs in the Genome of Drosophila Melanogaster Strain W1118; Iso-2; Iso-3. Fly.

[21] Afgan E., Baker D., Batut B., Van Den Beek M., Bouvier D., Ech M., Chilton J., Clements D., Coraor N., Grüning B.A. (2018). The Galaxy Platform for Accessible, Reproducible and Collaborative Biomedical Analyses: 2018 Update. Nucleic Acids Res..

[22] Gao J., Aksoy B.A., Dogrusoz U., Dresdner G., Gross B., Sumer S.O., Sun Y., Jacobsen A., Sinha R., Larsson E. (2013). Integrative Analysis of Complex Cancer Genomics and Clinical Profiles Using the CBioPortal. Sci. Signal..

[23] Weinstein J.N., Collisson E.A., Mills G.B., Shaw K.R.M., Ozenberger B.A., Ellrott K., Sander C., Stuart J.M., Chang K., Creighton C.J. (2013). The Cancer Genome Atlas Pan-Cancer Analysis Project. Nat. Genet..

[24] Cerami E., Gao J., Dogrusoz U., Gross B.E., Sumer S.O., Aksoy B.A., Jacobsen A., Byrne C.J., Heuer M.L., Larsson E. (2012). The CBio Cancer Genomics Portal: An Open Platform for Exploring Multidimensional Cancer Genomics Data. Cancer Discov..

[25] Subramanian A., Tamayo P., Mootha V.K., Mukherjee S., Ebert B.L., Gillette M.A., Paulovich A., Pomeroy S.L., Golub T.R., Lander E.S. (2005). Gene Set Enrichment Analysis: A Knowledge-Based Approach for Interpreting Genome-Wide Expression Profiles. Proc. Natl. Acad. Sci. USA.

[26] R Core Team R Core Team (2021). R: A Language and Environment for Statistical Computing.

[27] Gu Z. (2022). Complex Heatmap Visualization. iMeta.

[28] Gu Z., Eils R., Schlesner M. (2016). Complex Heatmaps Reveal Patterns and Correlations in Multidimensional Genomic Data. Bioinformatics.

[29] Fuchs T.L., Sioson L., Sheen A., Jafari-Nejad K., Renaud C.J., Andrici J., Ahadi M., Chou A., Gill A.J. (2020). Assessment of Tumor-Infiltrating Lymphocytes Using International TILs Working Group (ITWG) System Is a Strong Predictor of Overall Survival in Colorectal Carcinoma: A Study of 1034 Patients. Am. J. Surg. Pathol..

[30] Overman M.J., Lonardi S., Wong K.Y.M., Lenz H.J., Gelsomino F., Aglietta M., Morse M.A., Van Cutsem E., McDermott R., Hill A. (2018). Durable Clinical Benefit with Nivolumab plus Ipilimumab in DNA Mismatch Repair-Deficient/Microsatellite Instability-High Metastatic Colorectal Cancer. J. Clin. Oncol..

[31] Le D.T., Kim T.W., van Cutsem E., Geva R., Jäger D., Hara H., Burge M., O’Neil B., Kavan P., Yoshino T. (2020). Phase II Open-Label Study of Pembrolizumab in Treatment-Refractory, Microsatellite Instability–High/Mismatch Repair–Deficient Metastatic Colorectal Cancer: KEYNOTE-164. J. Clin. Oncol..

[32] Henry L.R., Lee H.O., Lee J.S., Klein-Szanto A., Watts P., Ross E.A., Chen W.T., Cheng J.D. (2007). Clinical Implications of Fibroblast Activation Protein in Patients with Colon Cancer. Clin. Cancer Res..

[33] Liu R., Li H., Liu L., Yu J., Ren X. (2012). Fibroblast Activation Protein: A Potential Therapeutic Target in Cancer. Cancer Biol. Ther..

[34] Greimelmaier K., Klopp N., Mairinger E., Wessolly M., Borchert S., Steinborn J., Schmid K.W., Wohlschlaeger J., Mairinger F.D. (2023). Fibroblast Activation Protein-α Expression in Fibroblasts Is Common in the Tumor Microenvironment of Colorectal Cancer and May Serve as a Therapeutic Target. Pathol. Oncol. Res..

[35] Özdemir B.C., Pentcheva-Hoang T., Carstens J.L., Zheng X., Wu C.C., Simpson T.R., Laklai H., Sugimoto H., Kahlert C., Novitskiy S.V. (2014). Depletion of Carcinoma-Associated Fibroblasts and Fibrosis Induces Immunosuppression and Accelerates Pancreas Cancer with Reduced Survival. Cancer Cell.

[36] Rhim A.D., Oberstein P.E., Thomas D.H., Mirek E.T., Palermo C.F., Sastra S.A., Dekleva E.N., Saunders T., Becerra C.P., Tattersall I.W. (2014). Stromal Elements Act to Restrain, Rather than Support, Pancreatic Ductal Adenocarcinoma. Cancer Cell.

[37] Hofheinz R.D., Al-Batran S.E., Hartmann F., Hartung G., Jäger D., Renner C., Tanswell P., Kunz U., Amelsberg A., Kuthan H. (2003). Stromal Antigen Targeting by a Humanised Monoclonal Antibody: An Early Phase II Trial of Sibrotuzumab in Patients with Metastatic Colorectal Cancer. Onkologie.

[38] Scott A.M., Wiseman G., Welt S., Adjei A., Lee F.T., Hopkins W., Divgi C.R., Hanson L.H., Mitchell P., Gansen D.N. (2003). A Phase I Dose-Escalation Study of Sibrotuzumab in Patients with Advanced or Metastatic Fibroblast Activation Protein-Positive Cancer. Clin. Cancer Res..

[39] Fabre M., Ferrer C., Domínguez-Hormaetxe S., Bockorny B., Murias L., Seifert O., Eisler S.A., Kontermann R.E., Pfizenmaier K., Lee S.Y. (2020). OMTX705, a Novel FAP-Targeting ADC Demonstrates Activity in Chemotherapy and Pembrolizumab-Resistant Solid Tumor Models. Clin. Cancer Res..

[40] Zana A., Puig-Moreno C., Bocci M., Gilardoni E., Di Nitto C., Principi L., Ravazza D., Rotta G., Prodi E., De Luca R. (2023). A Comparative Analysis of Fibroblast Activation Protein-Targeted Small Molecule-Drug, Antibody-Drug, and Peptide-Drug Conjugates. Bioconjug. Chem..

[41] Lindner T., Giesel F.L., Kratochwil C., Serfling S.E. (2021). Radioligands Targeting Fibroblast Activation Protein (FAP). Cancers.

[42] Xu M., Chen J., Zhang P., Cai J., Song H., Li Z., Liu Z. (2023). An Antibody-Radionuclide Conjugate Targets Fibroblast Activation Protein for Cancer Therapy. Eur. J. Nucl. Med. Mol. Imaging.

[43] Kiani M., Jokar S., Hassanzadeh L., Behnammanesh H., Bavi O., Beiki D., Assadi M. (2024). Recent Clinical Implications of FAPI: Imaging and Therapy. Clin. Nucl. Med..

[44] Coto-Llerena M., Ercan C., Kancherla V., Taha-Mehlitz S., Eppenberger-Castori S., Soysal S.D., Ng C.K.Y., Bolli M., von Flüe M., Nicolas G.P. (2020). High Expression of FAP in Colorectal Cancer Is Associated with Angiogenesis and Immunoregulation Processes. Front. Oncol..

[45] Hintz H.M., Cowan A.E., Shapovalova M., LeBeau A.M. (2019). Development of a Cross-Reactive Monoclonal Antibody for Detecting the Tumor Stroma. Bioconjug. Chem..

