# Supplementary material for: Cancer-Associated Fibroblast Proteins as Potential Targets against Colorectal Cancers
Source: Cancers (Basel). 2024 Sep 14;16(18):3158. doi: 10.3390/cancers16183158 (PMC11440114; doi:10.3390/cancers16183158)
Supplement: Supplementary file 1 [file cancers-16-03158-s001.zip › Supplementary Captions.pdf]

Supplemental Materials Captions

Figure S1. Representative staining and scoring for collagen, MMP2, PDPN, and TAGLN in CRC patient tissues across tissue microarrays. Scale bar = 50 μm.

Figure S2. Heatmaps depicting percentage of all CRC patients with marker expression score in their primary CRC and marker expression in adjacent normal colon.

Figure S3. Heatmaps depicting percentage of all mCRC patients with a marker expression score in their CRC metastases to liver and marker expression in adjacent normal liver.

Table S1. Patient Demographics
