# Supplementary material for: Cancer-Associated Fibroblast Proteins as Potential Targets against Colorectal Cancers
Source: Cancers (Basel). 2024 Sep 14;16(18):3158. doi: 10.3390/cancers16183158 (PMC11440114; doi:10.3390/cancers16183158)
Supplement: Supplementary file 1 [file cancers-16-03158-s001.zip › Supplementary Table S1.pdf]

Table S1. Patient Demographics

Patient Demographics (n=243)	
**Age**	
EAO, <50 years old (%)	85 (35.0)
LAO, ≥50 years old (%)	158 (65.0)
**Gender**	
Male (%)	150 (61.7)
Female (%)	93 (38.3)
**Stage**	
Stage I (%)	34 (14.0)
Stage II (%)	31 (12.8)
Stage III (%)	48 (19.8)
Stage IV (%)	130 (53.5)
**Disease Site**	
Left Colon (%)	79 (32.5)
Right Colon (%)	82 (33.7)
Rectal (%)	71 (29.2)
Not Otherwise Specified (%)	11 (4.5)
